# Short-Term Functional Outcomes of Short Femoral Neck Stems Are the Same as Those of Conventional Stems in Primary Total Hip Arthroplasty

**DOI:** 10.3390/ijerph19084670

**Published:** 2022-04-13

**Authors:** Rafał Tkacz, Dariusz Larysz, Rafał Przybylski, Marta Tkacz, Krzysztof Safranow, Maciej Tarnowski

**Affiliations:** 1 Department of Trauma and Orthopaedic Surgery, 109 Military Hospital, 71-442 Szczecin, Poland; dariuszlarysz@hotmail.com; 2 Department of Orthopaedic Surgery, Independent Public Health Care Ministry of Internal Affairs and Administration, 70-382 Szczecin, Poland; ortoraf@gazeta.pl; 3 Department of Physiology, Pomeranian Medical University, 70-111 Szczecin, Poland; tkacz.mag@gmail.com (M.T.); maciejt@pum.edu.pl (M.T.); 4 Department of Biochemistry and Medical Chemistry, Pomeranian Medical University, 70-111 Szczecin, Poland; chrissaf@mp.pl

**Keywords:** osteoarthritis, total hip arthroplasty, hip implant, short stem

## Abstract

(1) Background: In this study, two types of implants were compared—a conventional hip stem and a femoral neck prosthesis. (2) Methods: The femoral neck prosthesis study group included 21 patients, while the conventional hip stem control group was 40 patients. The first examination was the pre-op check, while the next ones were performed 6 weeks, 1 year, and 3 years after surgery. The Harris Hip Score (HHS), Western Ontario and McMaster Universities Osteoarthritis Index (WOMAC), Oxford Hip Score (OHS), University of California at Los Angeles Activity Score (UCLA), and Visual Analog Scale EQ (VAS EQ) forms were completed at each clinical study visit. (3) Results: The HHS in the femoral neck prosthesis group and the conventional hip stem group 6 weeks after surgery was 68.8 ± 16.47 and 67.6 ± 8.92, respectively, and 1 year after surgery, this was 93 ± 5.58 vs. 90.6 ± 5.17, respectively. The OHS of the femoral neck prosthesis group was 34.8 points after 6 weeks, 45.5 points after 1 year, and 43.9 points after 3 years. The respective values in the conventional hip stem group were 35.5, 41.55, and 42.13 points. The WOMAC values for the femoral neck prosthesis group were 70.6, 92.7, and 86 points, respectively, while for the conventional hip stem group, they were 74, 88.1, and 86.1 points. The UCLA scores recorded in the conventional hip stem group ranged from 3.15 to 5.05 points, but a higher mean value of 5.33 points was obtained in the femoral neck prosthesis group. VAS EQ was equal to 84 points three years after the operation. (4) Conclusions: The study showed no significant differences in the functional scores of both groups, and the new type of cervical femoral stem could be the first choice in younger patients.

## 1. Introduction

Globally, of the 291 most common diseases, osteoarthritis (OA) of the knees and hip was ranked 11th among the causes of permanent disability and 38th among the diseases causing the longest number of days of disability [1]. The incidence of osteoarthritis of the hip is approximately 24/100,000, and the prevalence is 850/100,000. In the world population, the prevalence rate among women increases with increasing life expectancy [2]. Environmental factors are responsible for the largest proportion of cases of primary osteoarthritis of the hip joint [3], while genetic factors play a smaller role in the overall number of cases [4]. Intensive research over the last 20 years has significantly changed the view of the OA pathomechanism.

The incidence of OA increases with the average life expectancy of the population. As a result of damage to the cartilage within joints, including the hip joint, pain increases and mobility becomes restricted. Common symptoms of OA are crepitations, reduced mobility, and joint pain. The degenerative disease can also affect young, biologically active people. The main cause of hip osteoarthritis (HOA) is cartilage damage as a result of trauma, repeated microtraumas, repeated joint overload, and excessive wear associated with aging [5]. These processes may lead to changes in the composition, structure, and proportion of components in the cartilage tissue [6]. Hip arthroplasty restores fitness but also imposes some limitations, which are gradually being eliminated with the advancement of technology and surgical techniques. Total hip arthroplasty (THA) is one of the most common orthopaedic procedures performed in the world at present [7]. There is a wide selection of implants on the market, both cemented and fixed without bone cement. They differ, among other things, in the length of the stem, the material from which they are made, or the method of attachment to the bone. The widespread use of total hip arthroplasty in the treatment of osteoarthritis requires a reasonable choice of implants for individual patient groups. The extent of surgery may affect the function of the hip joint after surgery.

In this study, two types of implants were compared: a conventional cementless implant (conventional hip stem) and a modern femoral neck prosthesis. The femoral neck prosthesis was designed for patients requiring total hip arthroplasty with a well-preserved femoral neck. The implantation techniques for the investigated stems require different amounts of bone resection. The femoral neck prosthesis implant requires resection of the sub-capital fragment of the femoral neck, while the conventional hip stem requires subtotal resection of the femoral neck. Two parameters affect the assessment of the implant: in the short term, the functionality of the hip joint is the most important, and in the long term, the survival of the implant is most important. This paper assesses the functionality in a 3-year follow-up, providing a basis for further evaluation in terms of implant survival.

## 2. Materials and Methods

### 2.1. Clinical Materials

The patients were operated on Department VI of the Trauma and Orthopaedic Surgery of the hospital in Szczecin-Zdunowo, Poland. The femoral neck prosthesis study group was operated between 2012 and 2015, while the conventional hip stem control group consisted of patients operated on in 2013. All patients gave informed written consent to the implantation of the hip prosthesis. The consent for the clinical trial was granted by the District Medical Chamber 10/KB/V/2014. The study group included 21 patients, while the conventional hip stem control group consisted of 40 cases. The clinical characteristics of both groups of patients with osteoarthritis of the hip joint are presented in Table 1.

Each patient included in the study was informed about four meetings in which self-assessment forms and a clinical examination should be completed. The first examination was performed before the surgical procedure, while the next were performed 6 weeks, 1 year, and 3 years after the operation. The Harris Hip Score (HHS) form was completed at each clinical visit. In the interview, information was collected about the painkillers taken and smoking status. The Western Ontario and McMaster Universities Osteoarthritis Index (WOMAC), Oxford Hip Score (OHS), the University of California at Los Angeles Activity Score (UCLA) and Visual Analog Scale EQ (VAS EQ) self-assessment forms were completed by the patients themselves in the presence of an investigator. In case of doubts, patients could ask questions before selecting an answer. The interview, clinical examination, and filling in the forms took approximately 30 min. The data were computerized for further analysis.

### 2.2. Patient Selection

**Figure 1:**
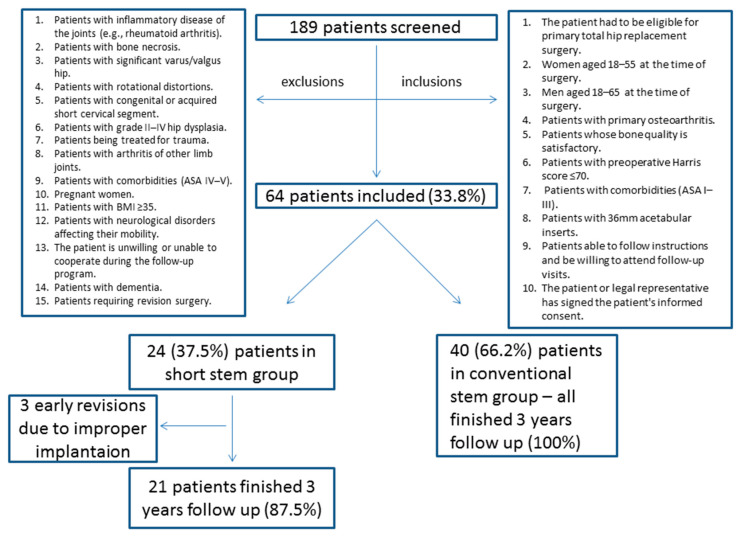
STROBE diagram detailing the inclusion and exclusion criteria.

The same inclusion and exclusion criteria were used for both groups (Figure 1). The conventional hip stem control group consisted of patients who did not consent to the femoral neck prosthesis implantation during the study period. The criteria were defined in order to optimise the assessment of the results of hip joint functionality. The main objective of the recruitment was to maximally reduce the effect of external factors, which can affect the results. 

### 2.3. Implants

**Figure 2:**
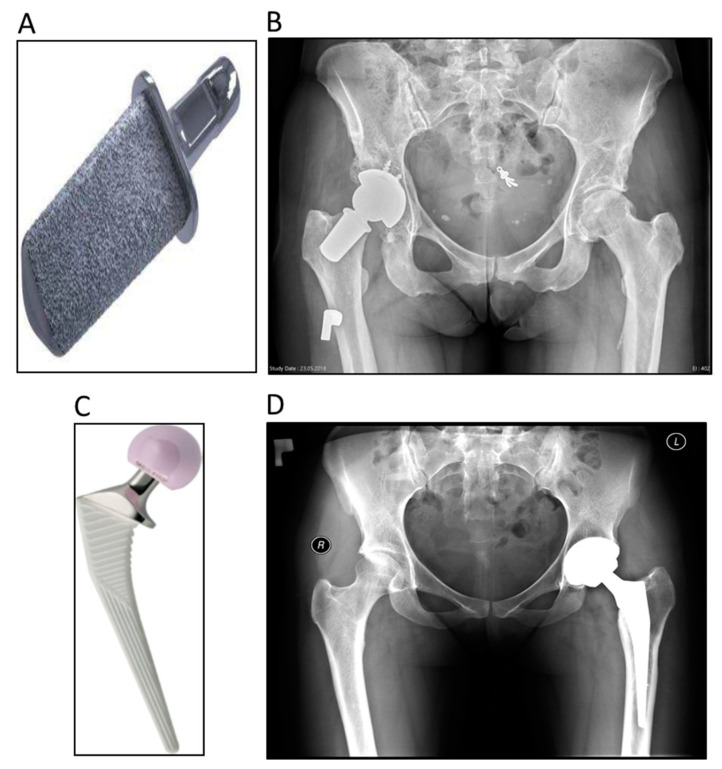
The femoral neck prosthesis (Biomet information materials) (**A**) and postoperative radiograph (**B**). The conventional hip stem system (DePuy information materials) (**C**) and postoperative radiograph (**D**).

The Primoris™ Femoral Neck (Figure 2A,B) is designed for patients requiring total hip arthroplasty and is dedicated to patients with a well-preserved femoral neck (a not well-preserved femoral neck is defined by 2–7 of the exclusion criteria in Figure 1). The endoprosthesis is made of an alloy of titanium, aluminium, and vanadium (TiAl6V4) and the surface is covered with hydroxyapatite (HA). The cone has a rectangular shape and is available in two lengths. The prosthesis is implanted without the use of bone cement. The cone is adapted with ceramic heads. The acetabular component was a Biomet Exceed cup with a ceramic 36-millimetre insert.

The Corail^®^ system (DePuy Synthes Raynham, MA, USA) (Figure 2C,D) was introduced to the market in 1986 [8]. Since then, this HA-coated cementless rectangular mandrel has become the gold standard among rectangular mandrels because of its excellent clinical results [9]. The acetabular component was a DePuy Pinnacle merged with a ceramic 36-millimetre insert. It is made of TiAl6V4 alloy. The proximal part expands in the sagittal and frontal sections, ensuring good fixation in the proximal metaphysis region of the femur. The distal part tapers downwards, providing good filling of the femoral canal. The stem has two-plane grooves to improve the mechanical stability of the implant. The HA coating prevents the release of metal ions from the stem and ensures good ossteointegration. The HA layer has a thickness of 150 µm and is applied by the plasma method.

### 2.4. Surgical Procedure

The standard Hardinge lateral access approach was used by dissection of the gluteal muscles. The mean duration of the operation was 57 min in the femoral neck prosthesis group and 48 min in the conventional stem group. In both types of operations, “press fit” cementless cups were used. The sockets and pins were coated with HA. 

### 2.5. Statistical Analysis

Since most of the analyzed quantitative variables showed significantly different distributions to the normal distribution (*p* < 0.05, Shapiro-Wilk test), non-parametric tests were used. The Mann–Whitney U-test was used for statistical and comparative analysis of the collected data, and Spearman’s rank correlation coefficient was measured. The obtained results were statistically analysed and expressed as arithmetic means with standard deviations, or presented graphically as box-and-whiskers plots showing median, quartiles along with the minimum and maximum. *p* values less than or equal to 0.05 were considered statistically significant. The statistical power of our study with 21 and 40 patients in the study and control group, respectively, was sufficient to detect, with 80% probability, the true effect size corresponding to differences in quantitative parameters between groups equal to ±0.8 of their standard deviations. Statistical calculations and visualization were carried out with commercial software (Statistica v. 13. Statasoft, Warsaw, Poland).

## 3. Results

### 3.1. Surgery-Related Complications

We noted three cases of early aseptic loosening of the femoral neck stem due to technical errors during implantation, and these were were excluded from the assessment of the functional scores. We did not notice any other complications in both groups. Observations of the desired groups continued.

### 3.2. Comparison of Groups before Surgery

The data collected from 21 patients in the femoral neck prosthesis group and 40 patients in the conventional hip stem group with the HHS, OHS, WOMAC, UCLA, and VAS EQ-5D forms before surgery in both groups are presented in Table 2.

### 3.3. Harris Hip Score (HHS)

**Figure 3:**
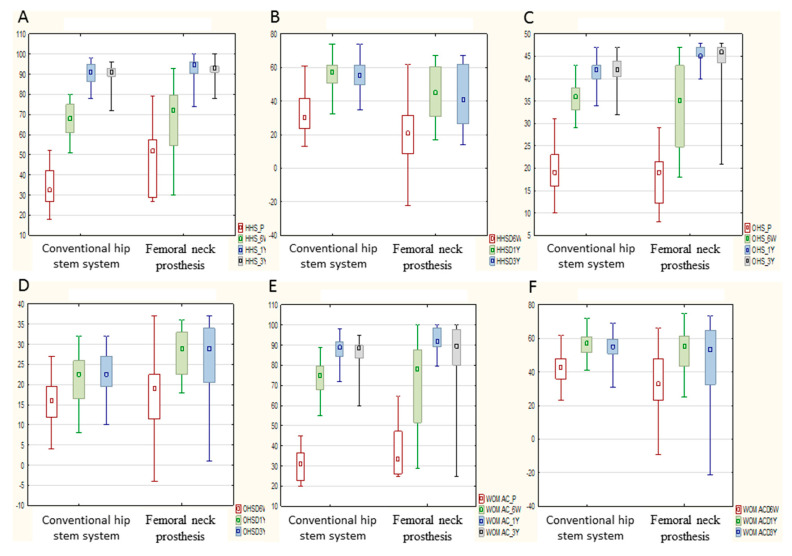

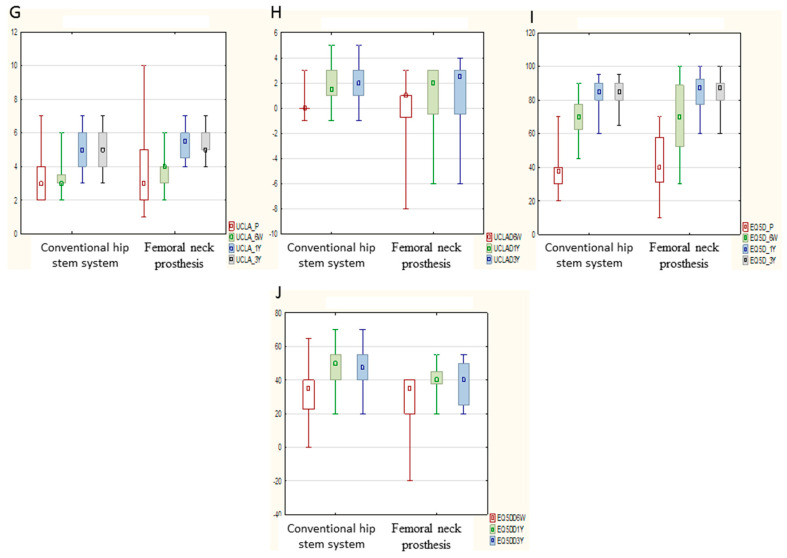
Box and whiskers plot graphs presenting median (point), quartiles (box), minimum and maximum (whiskers) in the functionality of the examined hips from HHS (**A**), HHSD (**B**), and various self-assessment forms: OHS (**C**), OHSD (**D**), WOMAC (**E**), WOMACD (**F**), UCLA (**G**), UCLAD (**H**), VAS EQ-5D (**I**), VAS EQ-5DD (**J**).

The HHS form was completed by the attending physician at each visit. Figure 3A shows the HHS values along with standard deviations and maximum and minimum values from the HHS forms from the follow-up visits, illustrating the improvement in function in an objective examination of patients.

There was a noticeable increase in scores for both groups during the observation. The biggest difference between the femoral neck prosthesis test group and the conventional hip stem control group was noticeable 6 weeks and 1 year after surgery, with 68.8 (SD 16.47) and 93 (SD 5.58) points (*p* ≤ 0.05), and 67.6 (SD 8.92) and 90.6 (SD 5.17) points (*p* ≤ 0.05), respectively. The next timepoint (3 years) showed a slight decrease in the HHS value in both groups: 92.7 (femoral neck prosthesis) and 89.9 points (conventional hip stem). The difference between the two studied groups was statistically significant (*p* ≤ 0.05). Figure 3B shows the increase in HHSD score compared to the preoperative score. There were two point-increments over the tested period: the first was 6 weeks after the surgery, with an average of 21.2 points for the femoral neck prosthesis test group and 32.9 points for the conventional hip stem control group; the second was a year after the surgery, with an average of 45 points for the femoral neck prosthesis group and 56 points for the conventional hip stem group. 

In the femoral neck prosthesis test group, the dispersion of scores was noticeably greater than in the control group. There was a moderate negative correlation with age in the HHS scores one year after THA (*p* ≤ 0.05). In the conventional hip stem patient group, there was a moderate negative correlation between BMI and HHS score one year (*p* ≤ 0.05) and three years (*p* ≤ 0.05) after surgery. One year after surgery, HHS scores were significantly lower in the group of non-smoking conventional hip stem patients (*p* ≤ 0.05).

### 3.4. Oxford Hip Score (OHS)

A patient’s self-assessment often differs from an objective assessment by a physician. The first tool for self-assessment of patients was the OHS form, which has a maximum score of 48 points; the higher the score, the better the patient’s self-esteem in terms of hip function. Figure 3C shows the scores collected from the OHS form together with the standard deviation as well as the maximum and minimum values. Once again, there is a noticeable two-stage increase in the score and a greater dispersion of values in the test group. The average score of the femoral neck prosthesis test group was 34.8 points after 6 weeks, 45.5 points after 1 year, and 43.9 points after 3 years. In the conventional hip stem group, the respective values were 35.5, 41.6, and 42.1 points. This tendency also persisted when the data were related to the preoperative score (Figure 3D). Regarding statistically significant results, there was a moderate negative correlation with age in the conventional hip stem patient group before surgery (*p* ≤ 0.05) and one year (*p* ≤ 0.05) and three years (*p* ≤ 0.05) after surgery. A similar trend exists in the femoral neck prosthesis group of patients, but it did not reach significance. The same dependence occurred in the correlation between BMI and OHS results in the conventional hip stem group after 6 weeks, (*p* ≤ 0.05) one year (*p* ≤ 0.05), and three years (*p* ≤ 0.05). No such trend was observed in the femoral neck prosthesis group.

### 3.5. The Western Ontario and McMaster Universities’ Osteoarthritis Index (WOMAC)

The WOMAC form is the most extensive questionnaire used in this study; the maximum score is 100 points. Figure 3E shows the dynamic progression of the scores within the first year after surgery (*p* ≤ 0.05); the values over the next two years did not significantly change. The values for the femoral neck prosthesis group were 70.6 (SD 21.1), 92.7 (SD 6.27), and 86 (SD 17.3) points. For the conventional hip stem group, they were 74 (SD 8.79), 88.1 (SD 5.66), and 86.1 (SD 6.57) points. Interestingly, the scores dropped after 3 years in relation to the values obtained one year after operation. The values in both groups were similar. The scoring dynamics graph (Figure 3F) is also similar. Once again, there is a greater dispersion of the score values in the femoral neck prosthesis test group. There was a negative correlation between age and WOMAC scores after one year (*p* ≤ 0.05), and three years (*p* ≤ 0.05). The same tendency can be seen in the evaluation of BMI vs. the WOMAC scoring after 1 year (*p* ≤ 0.05), and 3 years (*p* ≤ 0.05). There was no such trend in the femoral neck prosthesis group.

### 3.6. University of California at Los Angeles Activity Score (UCLA)

Figure 3G shows the point values from the form and Figure 3H shows the dynamics of the score increase. In the postoperative period, patients’ activity did not increase from baseline in both groups for the first 6 weeks. In the femoral neck prosthesis test group, there was even a decrease in the mean score by −0.23 points in relation to the score in the preoperative period. This can be explained by functional limitations for the first 4–6 weeks with the use of elbow crutches. A greater, statistically significant increase in UCLA scores was recorded in the conventional hip stem control group, from 3.15 to 5.05 points (*p* ≤ 0.05), but a higher value of 5.33 points was obtained in the femoral neck prosthesis test group. The difference after 3 years was not statistically significant between the two groups. In the conventional hip stem group, it was observed that the UCLA score decreased with the increasing age and BMI of patients (*p* ≤ 0.05). A similar tendency occurred in the femoral neck prosthesis group but did not reach statistical significance.

### 3.7. Visual Analogue Scale EQ-5D (VAS EQ-5D)

The general initial state of health was initially assessed as being better by patients from the femoral neck prosthesis test group compared to the conventional hip stem group, with 43.6 points vs. 36.8 points, respectively, probably due to the lower mean age of the entire study group. An increase in the mean score with time after surgery was seen in both groups. Figure 3I,J show the score and the score change from the preoperative value, along with the standard deviation and outliers. Both groups obtained 84 points three years after the operation. Due to the lower initial score of the conventional hip stem group, the score improvement in this group was greater. The moderate negative correlation of the VAS EQ-5D assessment with age reached significance in the femoral neck prosthesis group one year (*p* ≤ 0.05) and three years (*p* ≤ 0.05) after surgery. In the conventional hip stem group, there was a moderate negative correlation between the BMI and VAS in each study period (*p* ≤ 0.05).

### 3.8. Gender Differences

In the conventional hip stem group, women achieved significantly higher results in the control forms. Statistically significant differences were noted one year and three years after the operation (*p* ≤ 0.05). The femoral neck prosthesis test group included only four women, so the results are not conclusive. The results are presented in Table 3.

## 4. Discussion

### 4.1. Risk Factors in THA

Total hip arthroplasty is the most frequently performed orthopaedic procedure in the world. Universal access to educational materials, numerous courses, and practical training allows for a large group of operators to master the art of implanting hip implants. The currently available implants allow for the individualisation of the prosthesis selection process for individual patients and their requirements. When planning the primary treatment, it is always worth considering the need for potential revision treatments. Despite the good survival rate for implants, the loosening of components is the most common complication. The “fast track” protocol assumes a reduction in hospitalisation time, quick convalescence, and a reduction in the number of complications related to THA [10]. This approach requires the preoperative optimisation of risk factors that may worsen the prognosis after surgery. Two well-known risk factors are BMI and smoking habits [11]. This study confirmed a negative correlation between the assessment scores and BMI. Increased BMI makes initial rehabilitation difficult due to patients’ mobility problems. In the study, no single periprosthetic infection was observed, but the literature clearly shows that increased BMI is a significant risk factor for the development of infection after surgery [12]. On the other hand, a positive correlation between smoking and point scores was shown (data did not reach statistical significance). Smoking is a well-documented risk factor for both early and late complications after arthroplasty [13]. 

There was a noticeable correlation between the results and gender in both study groups. Women scored higher at the end of the study in both treatment groups, although the femoral neck prosthesis group scores did not reach statistical significance, due to the small sample size (only four women) (Table 3). Bheeshma Ravi presented similar observations during the AOOS conference in 2015 [14], noting that men are more problematic patients—they return to the emergency room more often, have a higher risk of revision in the first two years, and are more likely to be re-admitted to the hospital for a variety of orthopaedic reasons. This has a definite impact on the final score in the study.

### 4.2. Funcional Outcomes

Recommendations regarding the gradual return to activity are reflected in the test results. It is particularly visible during the follow-up visit carried out 6 weeks after the surgery. The slow increase in the scores in the HHS form illustrates this well (Figure 3B). The final HHS scores in the femoral neck prosthesis group were slightly higher than those of the conventional hip stem control group. This may be due to the lower average age of the cervical implant group. This was confirmed by the negative correlation between age and HHS score. Patients’ self-assessment of physical activity in the UCLA form was the most problematic during the study. The long waiting time for THA surgery in Poland often forces patients to work in a full-time job for a long period of time, despite pain and functional limitations. The drop in UCLA scores after surgery is related to elbow crutch mobility issues. These results do not differ from those published in the literature [15]. There were no statistically significant differences in the speed of rehabilitation during postoperative period between the study group and the control group (expressed as the dynamics of the score increment in the score forms). Huo et al. presented a meta-analysis of five randomised clinical trials involving 552 patients [16]. The authors considered the latest publications assessing the functional effects of THA. There is a significantly disproportionate difference between ours HHS assessment after 6 weeks in the study group and that from the meta-analysis. There was no such difference in the WOMAC self-assessment questionnaire. The meta-analysis included studies from Korea, Germany, Sweden, and Canada. The different model of patient care after THA in these countries somewhat explains the significant discrepancies in the fairly objective HHS results after 6 weeks. Our patients obtained lower scores, mainly due to insufficient mobility in the hip joint, which also affected the range of possible motor activities. The scores after one year were similar in both the meta-analysis and our study. This proves that, during convalescence, patients in our study compensated for the later shortcomings of hip joint mobility. On the other hand, the immediate analgesic effect of THA significantly improved the patients’ well-being, which can be explained by the high results in the WOMAC questionnaire (Figure 3E). There was a symmetrical increase in VAS EQ-5D self-esteem scores (Figure 3J) in both study groups, by about 30 points, 6 weeks after the operation, despite patients’ walking with two elbow crutches.

### 4.3. Physical Activity

The aim of the hip joint prosthesis is to restore full efficiency, as expected by patients. Standard recommendations for physical activity after THA advise against sports with a long and repetitive flight phase; the best example of this type of activity is running. Due to the progress in the design of implants, it is currently believed that, after surgery, the patients should not limit their sport-related activities, and the problem of potentially faster implant wear can be solved by revision surgery [17]. The results of the UCLA activity assessment used in the study show that some of the patients undertook sports activities described as moderate after THA—mainly cycling and swimming. Improving mobility and self-serviceability causes a significant increase in life satisfaction, increasing the well-being to similar levels to healthy people. The study confirmed that younger patients were more likely to take up sports activities after surgery.

### 4.4. Revision Surgery

The increasing amount of primary THA, the aging of societies, and the increasing number of young people undergoing THA cause a symmetrical increase in the number of hip revision surgery procedures (hip realloplasty). An increase in the number of primary procedures by 174% is predicted for the next 10 years, and the number of revision operations is expected to double [18]. Three basic risk factors of realloplasty within 12 years of THA were also determined: young age, high body weight, and patients with cemented prosthesis stems. The longer survival of endoprostheses with uncemented stems was also demonstrated in the retrospective study by Hailer et al. based on the excellent Swedish registry of arthroplasty [18]. 

Bone density studies one year after femoral neck prosthesis implantation showed only a slight decrease in bone mass in the femoral neck compared to the state before surgery. It is very promising in terms of the retention of this type of implant [19]. A study on the use of short-stem implants showed a 90% chance of 15-year implant survival [20]. Problems with bone density in the femoral neck were mainly related to the use of oral steroids. The follow-up period in our study was short (3 years), and it was difficult to clearly assess the durability of the femoral neck prosthesis implants. The preliminary data are promising and indicate good mechanical strength and correct osseointegration of the femoral neck prosthesis implants. 

**Figure 4:**
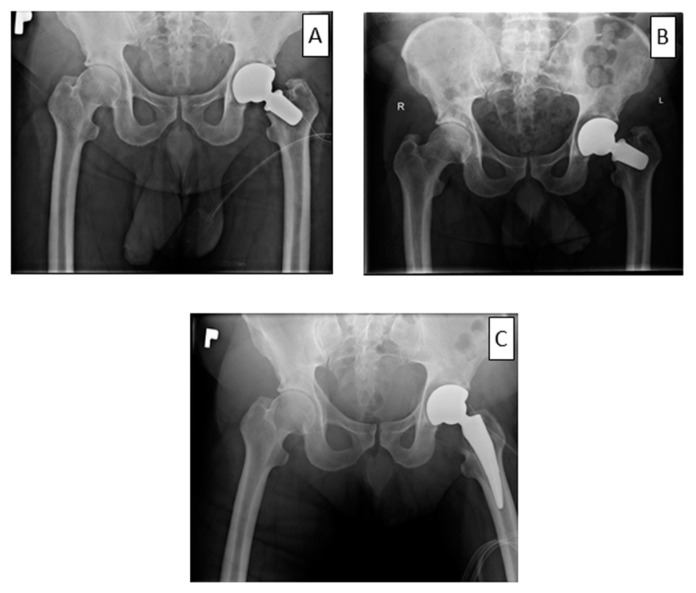
The case of early loosening from the short-stem group. X-ray taken after implantation (**A**), loosening 4 weeks after implantation (**B**) and after revision surgery (**C**).

Hip biomechanics after THA is usually affected. Implant survival is dependable on centre of the rotation, frictions and cup positioning. All these factors can lead to shortening of the implant survival [21]. Interesting experiences arose from three revisions performed on patients with a loosened femoral neck prosthesis implant shortly after the primary surgery. In all three cases, the implant was converted to an implant with a longer but conventional stem. The operations were problem-free and lasted for a relatively short time, as for a revision procedure (average 44 min). The operations required cutting the femoral neck and implementing a previously selected standard stem, which did not lead to any major technical problems. Oversized rasp, cementation, and augmentation with bone grafts were avoided. Blood loss was minimal, and none of the patients required a blood transfusion after surgery (Figure 4). Further work addressing the revision of standard and short mandrels is required to confirm these observations. In the opinion of the operator, it was easier to convert to a standard stem than to a revision implant. The decision regarding the choice of a short-stem prosthesis in younger people seems to be reasonable, mainly in the context of revision.

### 4.5. Limitations of the Study

This study shows that the functionality of the hip joint after insertion of this implant does not differ from one of the best-tested conventional hip stem implants. We are aware that our study has certain limitations. The groups of patients were relatively small and heterogeneous, and the observation time was too short to determine the superiority of the femoral implants.

## 5. Conclusions

The emerging new types of implants on the market increase the possibility of selecting equipment that is individualized to the needs of patients. The new type of femoral cervical stem is targeted for young patients with a proper density of the femoral neck. It provides a better anatomical distribution of physical forces within the hip joint after implantation. 

Limited bone resection during surgery does not significantly accelerate patients’ convalescence after surgery, but significantly facilitates revision surgery in the event of cervical implant loosening. Furthermore, we found that the final effects of the treatment were better in women than in men. This is consistent with the observations of researchers from other centres. The increased needs of patients after THA require new planning for implant selection. The increase in sports activity may increase the number of revision operations required in the coming years. This is of particular importance in the youngest patients undergoing THA. During their entire life, they may have to undergo two or even three revision operations due to increased physical activity. The use of a cervical implant gives the patient greater comfort with an active lifestyle. Moreover, it provides more opportunities for revision in case of loosening from excessive use. In the event of a revision, the bone outside the femoral neck remains intact, allowing for the use of another short implant (for example, metaphyseal). Short-stem prosthesis could be recommended as a first-choice implant for patients under 50 years old, mainly in the context of revision.

To date, we have not recognised any loosening of the implant in both groups, and the patients are still doing well. Future investigations should be conducted, mainly in the context of revision surgery after the use of short-stem implants.

## Figures and Tables

**Table 1:** Clinical characteristics of the studied groups.

Clinical Data	Femoral Neck Prosthesis (*n* =21)	Conventional Hip Stem (*n* = 40)	*p*
Age (year, average) (±)	50.57 (SD 10.6)	55.34 (SD 7.25)	0.21824
Male	17 (80.9%)	25 (62.5%)	
Female	4 (19.1%)	15 (37.5%)	
BMI	25.57 kg/m^2^ (SD 3.74)	26.97 kg/m^2^ (SD 4.13)	0.21542
Smoking	33%	17.5%	0.18853

**Table 2:** Pre-operative form assessments.

Form/Max Score	Femoral Neck Prosthesis Group (±SD)	Conventional Hip Stem Control Group (±SD), *p*-Value
HHS/100	47.57 (15.7)	34.63 (8.87), *p* 0.0018
OHS/48	16.66 (5.9)	19.57 (5.35), *p* 0.22
WOMAC/100	37.66 (11.99)	31.18 (7.56), *p* 0.049
UCLA/10	3.9 (2.6)	3.1 (1.19), *p* 0.62
VAS EQ-5D/100	43.57 (18.32)	36.81 (10.36), *p* 0.04
Smokers	7	6

**Table 3:** Gender differences.

	Women Conventional Hip Stem	Women Femoral Neck Prosthesis	Men Conventional Hip Stem	Men Femoral Neck Prosthesis
HHS 1Y	**94.23**	95.5	**88.74**	92.36
HHS 3Y	**92.23**	94.5	**88.71**	92.21
OHS 1Y	**43.07**	45.5	**40.76**	45.5
OHS 3Y	**44.07**	44.25	**41.12**	43.79
UCLA 1Y	**5.69**	5.75	**4.64**	5.21
UCLA 3Y	**5.92**	5.25	**4.6**	5.43
WOMAC 1Y	**90.76**	92.05	**86.72**	92.91
WOMAC 3Y	**90**	89.9	**84.84**	84.92

Bolded *p* ≤ 0.05.

## Data Availability

The data presented in this study are available on request from the corresponding author.
